# Perspectives in Pancreatic Pain

**DOI:** 10.1155/1997/46108

**Published:** 1997

**Authors:** A. S. Salim

**Affiliations:** Department of Surgery National University of Malaysia Jalan Raja Muda Kuala Lumpur Malaysia

## Abstract

This review describes some of the mechanisms which are thought to be important in the causation of pain in chronic pancreatitis. Both medical and surgical techniques for treating this pain are described.


HPB Surgery, 1997, Vol. 10, pp. 269–277
Reprints available directly from the publisher
Photocopying permitted by license only

© 1997 OPA (Overseas Publishers Association)
Amsterdam B.V. Published in The Netherlands
by Harwood Academic Publishers
Printed in India

### Perspectives in Pancreatic Pain

A. S. SALIM

*Department of Surgery, National University of Malaysia, Jalan Raja Muda, Kuala Lumpur, Malaysia*

*(Received 25 October 1993)*

**This review describes some of the mechanisms which are thought to be important in the causation of pain in chronic pancreatitis. Both medical and surgical techniques for treating this pain are described.**

*Keywords:* Chronic pancreatitis, pain control, surgery for chronic pancreatitis

### INTRODUCTION

Chronic pancreatitis is not a simple disease entity but an expression of multiple diseases such as alcoholism, tropical malnutrition, hyperparathyroidism, hyperlipidaemia, hereditary predisposition and ductal obstruction. Geographical differences in the incidence and natural history of this disease are clearly observed. In each case the anatomical deformities must be individually assessed with the operative choices and techniques, if indicated, remaining flexible and adaptable.

Chronic pancreatitis is characterized by both the persistence and the relentless progression of pancreatic lesions in contrast to acute pancreatitis where the pancreatic lesions usually resolve. The natural history of chronic pancreatitis is punctuated by recurring episodes of acute inflammation. According to the Marseilles classification, this has been termed relapsing or recurrent chronic pancreatitis, however it is customary nowadays to identify pancreatitis only as acute or chronic [1, 2]. The inflammatory process initially affects and destroys the exocrine tissue and then the islets of Langerhans, however the functional reserve capacity of the pancreas is so great that clinically significant pancreatic insufficiency does not occur until more than 85% of functioning tissue is lost [1]. Then malabsorption, steatorrhoea, and insulin-dependent diabetes ensue. Pancreatic calcification, usually evident on a plain upper abdominal radiograph, occurs in 30–50% of patients. Possible complications include malabsorption, weight loss, diabetes mellitus, ascites and/or pleural effusion, narrowing of the common bile duct, duodenum and/or colon, left-sided portal hypertension following thrombosis of the splenic, superior mesenteric and/or portal veins, major arterial haemorrhage, and pain [1]. Intractable abdominal pain is a feature in more than 95% of patients with chronic pancreatitis [1].

### THE PATHOLOGY OF CHRONIC PANCREATITIS

Chronic alcoholism is the most common cause of chronic pancreatitis in the Western World.

Because only about 10% of alchoholics develop the disease, it may be that a certain predisposing factor, perhaps a hereditary disorder exposed by an environmental or nutritional influence, increases susceptibility to the disease. The pancreatic juice in patients with alcohol-induced chronic pancreatitis has an additional protein, lactoferrin, which has a molecular weight of 69000 daltons and is slightly anionic at pH8.6 [1]. This protein is associated with the formation of acidic macromolecules and complexes which may be a key factor in the formation of the protein precipitates noted in the ducts of alcoholic patients with chronic pancreatitis [1]. The lesions of this disease may progress through a precalcifying stage to eventual clacification and stone formation [1]. Microscopically, the earliest changes appear to be precipitates of proteinaceous material in the intercalated and canalicular ductules. Later on calcium carbonate is added to the glycoprotein plugs leading to the characteristic calcifications, a process enhanced by the increased secretion of ionized calcium in the pancreatic juice of the alcohol-induced chronic pancreatitis patient [1]. The ductular obstruction caused by the protein plugs is associated with small ductular cavities lined by cuboidal epithelium and containing pancreatic juice, debris, necrotic matter or blood. In addition, the plugs cause ductular dilatation, which is followed by acinar atrophy [1]. A variable inflammatory infiltrate may occur in the interstitium with fibrosis within and between the lobules and around the ducts [1]. At the final stages, the pancreas is replaced by fibrous tissue with a few nests of acinar tissue and some islet cells [1]. The ducts are usually widely dilated with pseudosacculations and interspersed areas of dilatation and stenosis (a chain of lakes). It is not uncommon, however, to see normal ducts.

Obstructive chronic pancreatitis can be caused by fibrosis of the ampulla of Vater, papillary inflammation, congenital or acquired strictures of the main duct, and pancreatic paranchymal tumours. Following ductal obstruction, diffuse lesions with no lobular topography appear and spare no exocrine tissue [1]. The ductal caliber remains regular with no alternating stenosis and dilatation. The major ducts are dilated moderately, while the finer branches have normal diameters. The ductal epithelium is intact, the duct lumina are empty, and protein plugs as well as calcifications are rare [1]. Nevertheless, many patients with chronic pancreatitis have normal-sized or even narrowed ducts.

Surgical management of the disease is influenced by duct size [3]. Patients with ducts enlarged to 8 mm or more in diameter are considered for a drainage procedure to relieve pain. Conversely, patients with smaller ducts are candidates for resection. Moreover, there is some evidence that the natural history of the disease and the likelihood of complications may be different depending on whether the patient has small or large duct disease. Nealon and coworkers [4] described 93 patients with chronic pancreatitis (22 with small ducts and 71 with large ducts) who were evaluated prospectively. Of the 71 with large ducts, 43 underwent pancreaticojejunostomy. A total of 75 patients were assessed 14 months later. They concluded that patients with small duct chronic pancreatitis deteriorated functionally more slowly than those with unoperated large duct chronic pancreatitis. Those with large duct chronic pancreatitis who underwent operation did better than either of the other 2 groups.

About one third of patients with calcifying chronic pancreatitis develop jaundice from common duct compression at some time during their disease. Two clinical situations are recognized: (a) During an acute episode of pancreatic inflammation, the bile duct is compressed by the swollen head of pancreas. (b) In about 2% of cases jaundice is due to fibrotic narrowing of the common bile duct [5]. Most often there is a diffuse, longitudinal, symmetrical stricture of the intrapancreatic portion of the common bile duct with minimal dilatation of the duct proximally. The obstruction is almost never complete. The gallbladder and cystic duct may be dilated but usually much less than with cancer [5]. Less, commonly, there is an hourglass shaped narrowing localized at the upper border of the pancreas. This type is rarely associated with jaundice [5].

Cysts and pseudocysts are also seen in chronic pancreatitis [1]. True cysts are produced by ductal obstruction with continued pancreatic juice secretion into the duct. They start as dilatations of the pancreatic ductules which enlarge and break into the peripancreatic tissue forming ill-defined cystic collections surrounded by a reactionary sclerosis. At this stage such cysts are no longer lined by epithelium and are pseudocysts. With continued pancreatic necrosis there may rupture into the peripancreatic spaces forming a fibrous tissue lined cavity surrounded and limited by the neighboring organs.

Chronic pancreatitis may lead to duodenal and colonic obstruction [6]. The inflammatory pancreatic exudate containing active enzymes and other toxic substances reaches the duodenum and colon through their peritoneal and mesenteric attachments [6]. The duodenal fibrosis may also be due to ischaemia superimposed on inflammatory oedema. Many of the arteries and nerves supplying the duodenum pass first through pancreatic tissue and are susceptible to injury during pancreatic inflammation. The colonic involvement depends on the site bearing the brunt of chronic pancreatitis. Disease in the head and body of the pancreas may affect the transverse colon; disease in the tail of the pancreas may involve the splenic flexure. Histological examination of the obstructed segment of colon shows chronic inflammatory fibrosis and spread of inflammatory exudate to the vessels causing vascular thrombosis and colonic ischaemia.

Blood vessels are also affected by chronic pancreatitis where the smaller arteries within the gland become increasingly tortuous and beaded and the larger arteries outside the gland may be destroyed by activated proteolytic enzymes leading to haemorrhage. The veins are also involved and while splenic vein occlusion is usually recognized, involvement of the superior mesenteric and portal veins may be overlooked perhaps until unexpected varices are seen during laparotomy.

Chronic pancreatitis causes changes in the pancreatic nerves manifested by inflammation and destruction of the perineural sheath thus rendering the nerve exposed to irritants (pancreatic enzymes and kinins). These changes are particularly important due to their relationship to the mechanism of pain in chronic pancreatitis.

### PANCREATIC PAIN

Pain is a prominent feature of both acute and chronic pancreatitis as well as pancreatic cancer. The natural history of chronic pancreatitis is punctuated by recurring episodes of acute inflammation which are manifested by severe abdominal pain. This pain poses considerable management problems because it may prove to be difficult to treat and lead to narcotic addiction and significant psychological disturbances. Consequently, serious socio-economical problems become an added dimension in the management of these patients. The success of medical management of the pain may fail unless patients give up alcohol completely which may need the support of special clinics. Intractable pain may call for total pancreatectomy after the patient has completely given up alcohol, an operation which, in other than the most experienced hands, is likely to have an unacceptable mortality and morbidity [3, 7–9]. Any measure that can safely control, or at least reduce, pancreatitis-induced pain will have far reaching social and economical advantages besides obvious medical implications.

Pancreatitis-induced abdominal pain is thought to be multifocal in origin and most commonly caused by increased intraductal pressure, fibrosis or inflammation around peripancreatic nerves, or acute inflammatory episodes [7]. The role of intraductal pressure in the mechanism of pain was initially supported by intraoperative and later by endoscopic measurements. Japanese investigators using an endoscopic technique found higher intraductal pressure in patients with painful chronic pancreatitis than in those in whom it was painless [10]. It has also been established that a close correlation exists between tissue pressure and pain in patients with chronic pancreatitis who underwent drainage operations. A substantial reduction in pressure after drainage was followed by pain relief and this pressure was noted to increase once again when the pain recurred [8, 9]. The neural changes occurring in chronic pancreatitis (increase in the number of nerves in inflammatory pancreatic tissue and degeneration of the perineurium) allow an influx of inflammatory mediators and activated pancreatic enzymes to play a role in the mechanism of pain [1, 7, 8]. The possibility exists that the increased pressure and the neuritis operate synergistically to produce the pancreatitis-induced pain. The high tissue fluid pressure may facilitate influx of inflammatory mediators into the nerves. The acute episodes of pain that occur in the early stages of typical chronic pancreatitis might be related to recurrent autodigestive necrosis and enlargement of pseudocysts. Since the magnitude and duration of pain cannot be correlated with the morphological changes in the gland, it has been proposed that the pain may be associated with the dynamics of the disease rather than with the static situation [8, 9].

### MEDICAL MANAGEMENT OF PANCREATIC PAIN

The treatment of this symptom has not changed significantly over the past few years. Apart from insisting on total abstinence from alcohol, the treatment of pancreatic pain includes analgesia (narcotic or non-narcotic agents alone or with NSAIDs) with or without tricyclic anti-depressants, anti-neoplastic agents, prednisolone and pancreatic enzyme supplements [8]. These supplements relieve pain via the effect of trypsin on pancreatic secretion. The lack of trypsin activity within the intestinal lumen causes hyperstimulation of the gland by cholecystokinin, and perhaps also by cholinergic pathways, which might lead to high intraductal and tissue pressure and pain. Restoration of intestinal trypsin may enhance pain relief [8]. On the basis of the duct tissue-hypertension theory, endoscopic stenting of the main pancreatic duct in chronic pancreatitis, and the dorsal duct in pancreas divisum, has been used to relieve pain. This approach, however, fails to overcome outflow obstruction from secondary or tertiary ducts and is thereby unable to completely relieve the pain [11, 12].

Most of the sensory nerves to the pancreas pass through the coeliac ganglion and splanchnic nerves. In patients with pancreatitis whose exocrine and endocrine functions have already been compromised, nerve ablation procedures by the percutaneous injection of anaesthetic agents, ethanol, or phenol have been used to obtain pain relief [13]. The results of these procedures, however, have been disappointing with no more than half of the treated cases obtaining pain relief for about two months [13]. Conversely, coeliac plexus chemoneurolysis should be considered in pancreatic cancer more often than it is today, and at an earlier stage [8].

Regardless of the cause of episodes of pancreatic pain one of the feature of chronic pancreatitis is a continuous inflammatory process with round cell infiltration [1]. Since oxygen-derived free radicals are cytotoxic agents that produce tissue damage and inflammation [7, 14], and since these radicals are released by oxidative bursts from inflammatory cells [15], it may well be that they mediate the mechanism of pancreatic pain and its recurrent episodes. Nonaka *et al.* [16] confirmed that free radicals are a key factor in the development of acute pancreatitis in experimental animals [17] and reported that the imbalance between the offense system represented by xanthine oxidase and lipid peroxide and the defense system reflected by superoxide dismutase might be an important cause of tissue damage induced by free radicals. In man [7], administration of radical scavengers such as allopurinol and dimethyl sulphoxide (DMSO) significantly increased the efficacy of narcotic analgesics in the treatment of recurrent episodes of pancreatitis-induced pain and significantly reduced white cell counts and lactate dehydrogenase levels. The serum lactate dehydrogenase is a measurement of the amount of tissue destruction and may reflect the magnitude of pancreatic and peripancreatic inflammation and necrosis [18] and the white cell count is a measure of the local tissue reaction. One of the major gains attained by the use of radical scavengers was the accelerated discharge of patients from the surgical unit. Although DMSO directly scavenges hydroxyl radicals it also inhibits inflammatory cell functions [19]. These cells can participate in the process of tissue injury by oxidative bursts which yield reactive oxygen species [19] and since these cells also mediate the inflammation associated with the recurring episodes of chronic pancreatitis, the possibility exists that DMSO might have combated free radicals to a greater or lesser extent by inhibiting inflammatory cell functions.

A proportion of patients have an anxiety syndrome partly manifested as abdominal pain which may be controlled with appropriate support and anti-depressant treatment.

Results of pain control or the lack of it have to be based on objective parameters of assessment and in this regard linear analogue scales of pain severity are helpful.

### SURGICAL PROCEDURES FOR PAIN RELIEF

Surgery may help two thirds of the patients obtain pain relief provided the surgical procedure is carefully chosen. Thus, duct decompression operations are designed to reduce duct-tissue pressure while saving the paranchyma in patients with a dilated main pancreatic duct whereas resectional surgery removes the origin of pain by excising inflammatory, necrotic, fibrotic or cystic tissue. Surgery is therefore offered for symptomatic relief and not correction of glandular malfunction.

A distinction has to be made between obstructive chronic pancreatitis due to inflammatory or tumoural occlusion of the pancreatic ducts, which can be cured by treatment of the obstruction, and chronic calcifying pancreatitis resulting in irreversible sclerosis of the pancreatic paranchyma with or without dilatation of the ducts and the possible development of intrapancreatic pseudocysts, biliary or duodenal stenosis, and sometimes portal hypertension.

The natural history of chronic pancreatitis usually proceeds through four phases:

1. a latency period which may extend for many years.
2. a clinically-active stage lasting 3–5 years manifested by recurrent pain and acute crises with possible development of pseudocysts.
3. an intermediate stage lasting about 5 years during which the pain decreases in intensity and frequency.
4. a late stage where pain usually disappears but diabetes is found in 80% of the surviving cases.

Mortality in patients with chronic pancreatitis is higher than in the general population with 25–50% of cases dying 10 years after the clinical phase has commenced [20]. This aspect of the disease has to be taken into consideration in relation to the expected survival gain that might accure from operative intervention. Evaluation must include knowledge of the operative mortality, quality of functional results, and re-operation rate. As drug addiction is one of the disheartening consequences of severe pancreatic pain, surgery for the pain should be offered well before the patient becomes a narcotic addict and before the patient develops nutritional or metabolic disturbances. The weight of clinical evidence, however, does not lend support to the concept that pancreatic function improves following decompressive operations [9].

Nerve ablation procedures by surgical splanchnicectomy and coeliac ganglionectomy to interrupt sensory innervation of the pancreas are technically demanding due to access limitations and have been replaced by percutaneous chemoneurolysis but with very minimal gains. Surgical nerve ablation may, nonetheless, be considered as an alternative after a failed drainage or resection.

Operations on the sphincter of Oddi and the sphincter of Wirsung are suitable for those very rare cases that have a localized ampullary stenosis leading to a uniformly dilated pancreatic duct. It is unlikely that chronic pancreatitis is responsible for this stenosis which is probably a sequelae of complicated choledocholithectomy. It is possible, however, that endoscopic sphincterotomy in association with external lithotripsy to eliminate pancreatic stones may radicaly modify present treatment of chronic calcifying pancreatitis.

Pancreaticojejunostomy is well established as an effective ductal drainage procedure provided the main pancreatic duct is dilated. Side-to-side pancreaticojejunostomy is the method of choice because its longer anastomosis is thought to be crucial in providing long-term drainage and pain relief. This procedure has the advantage of saving pancreatic glandular tissue in addition to having low morbidity and mortality rates. The anastomosis should ideally be at least 8 cm long to avoid stenosis at a later date. In addition, retaining the spleen eliminates the risk of postsplenectomy sepsis. The proportion of patients becoming completely or partially pain-free averages 70% and this relates inversely to the length of the postoperative period, however the majority of cases still experience pain relief after 5 years [9]. This response is promoted by complete abstinence from alcohol intake.

It remains to be seen whether ductal drainage by pancreaticogastrostomy [21] offers any additional advantages in terms of pain relief over those already afforded by pancreaticojejunostomy.

Triple bypass, consisting of pancreaticojejunostomy with choledochojejunostomy and gastrojejunostomy, without pancreatic resection is associated with technical complexity and operative risk similar to those of pancreaticoduodenectomy and incurs more failures and reoperations. A review of this operation showed a mortality rate of 4.5% and after 2 years the survival rate was 78% with 85% of the cases having good functional results but 13% of the patients requiring reoperation [22]. Attempts to avoid marginal ulceration by biliary, pancreatic and duodenal diversion using interposition of an isolated jejunal loop between the pancreatic duct and the jejunum are yet to be confirmed independently [20].

Refinements of endoscopic techniques may enable a wider use in the future of endoscopic methods to treat chronic pancreatitis particularly cystogastrostomy for pseudocysts [20].

Resective procedures in chronic pancreatitis remove part or all of the pancreas to achieve pain relief. The best results of resection are seen when the head of the gland is removed in patients with major involvement of this part of the gland, intractable pain, and absence of dilated pancreatic ducts. The classical Whipple pancreaticoduodenectomy operation is still the most commonly used procedure under these circumstances, but other operations that preserve the stomach and duodenum have been introduced and detailed assessment of their exact role in the treatment of chronic pancreatitis-pain is eagerly awaited [8, 9]. The mortality and morbidity rates of the Whipple procedure continue to be high in other than experienced hands, thus it is recommended that the selection and treatment of patients must be carried out in specialty centres familiar with this type of surgery.

Left resections of the pancreatic gland have been suggested. The initial promising results of 80% pain relief reported by Fry *et al.* [23] following 95% left-sided pancreatic resection were, unfortunately, reduced to 56% after a mean follow-up of 7.8 years. A major additional problem was the high incidence of diabetes and late deaths (27%) mainly related to metabolic disturbances caused by the extent of resection. These disappointing results have also been noted by others [8, 9], thus there is little enthusiasm today to carry out this type of surgery. Pain is seldom the main reason for operation in pancreatic carcinoma, however the palliative value of pancreaticoduodenal resection must not be overlooked in patients who are not curable but have resectable primary lesions and a few metastases in the regional lymph-nodes and/or liver. Once again the emphasis has to be on carrying out this operation in specialist centres to reduce risks. Although total pancreatectomy aims to remove the source of pain, it still carries the risk of operative mortality with difficulties in endocrine control and may not succeed in affording complete pain relief [8,9]. Thus, the dismal results of this procedure and its high early and late morbidity restrict its application in patients with chronic pancreatitis.

### NEW SURGICAL APPROACHES

The results of surgical treatment must be judged primarily by the grade of pain relief measured against the risks entailed. With this in mind, there have been serious efforts to advance surgical contribution to pain relief in the chronic pancreatitis patient. Cuilleret and Guillemin [20] reported satisfactory results with the Gauthier-Benoit bipolar pancreatectomy where a pancreaticoduodenectomy is performed in patients who have previously undergone distal pancreatectomy preserving a small isthmic segment of the pancreas for anastomosis to the jejunal loop. The indications for this procedure remain exceptional and its exact role in the surgical management of pancreatic pain is still to be defined. The duodenum-preserving resection of the head of pancreas as described by Beger and colleagues [24] includes excision of the pancreatic head apart from a rim between the duodenum and bile duct at the inner curvature of the duodenum. The mesoduodenal vessels, and not necessarily the gastroduodenal artery, must be saved when resecting the uncinate process to preserve the duodenal blood flow. The resected end of the left pancreas is anastomosed to a jejunal Roux loop which is also connected to the small pancreatic remnant at the inner curvature of the duodenum. In 57 patients, the hospital mortality was 2% and 90% of these patients continued to have significant pain relief 2 years after surgery [24]. Frey and Smith [25] introduced a similar approach for patients with ductal dilatation and strictures and a markedly enlarged fibrotic pancreatic head. The duct in the corpus and cauda are opened and the pancreatic head is cored out, leaving a rim of glandular tissue along the inner aspects of the duodenum and the bottom of the crater. A Roux loop is anastomosed side-to-side to the opened pancreatic duct as well as to the cored out head of the gland. Frey and Smith reported 6 cases with an uneventful postoperative course [25]. The effect on pain is less clear. The satisfactory long-term effects and low morbidity of the pylorus-preserving pancreaticoduodenectomy and the good results with the duodenum-preserving total pancreatectomy in infants for nesidioblastosis led to the development of total pancreatectomy with preservation of the whole duodenum for advanced chronic pancreatitis [26]. Fourteen adult patients were operated on with no hospital mortality and no major post-operative complications. Good pain relief was noted in two-thirds of the patients besides improved gastrointestinal function and easier control of the diabetes [26]. Another operation for chronic pancreatitis is the denervated pancreatic flap operation [27]. Following subtotal resection of the pancreatic head the remaining pancreas is mobilized from its bed while vascularity is preserved via retrograde flow from the splenic hilus. Five patients underwent this procedure without mortality but 2 cases had major postoperative complications. At 9 months, 3 of the 5 patients had good pain control. Larger series of patients by various centers is a pre-requisite before these new surgical procedures can be adopted and gain a position among current surgical strategies.

### THE PRESENT POSITION

A consensus has been currently reached on the role of surgery in the treatment of chronic pancreatitis:

1. The natural history of the disease cannot be influenced by surgical treatment which must therefore play a palliative role with particular emphasis on pain relief and prevention of complications.
2. Total and subtotal pancreatectomy must be used selectively due to their operative mortality and their metabolic sequelae. Splanchnicectomy is no longer used alone for its results are too uncertain. Distal pancreatectomy is performed only when the disease is limited to the left part of the gland and even then many surgeons add a pancreaticojejunostomy.
3. Long-term survival and functional results depend mainly on alcohol withdrawal, which is a mandatory pre-requisite before any elective surgical treatment.

Current trends in Europe include performing fewer resections and more pancreaticojejunostomies than 10 years ago [20]. Nonetheless, pancreaticoduodenectomy, which has the advantage of treating or preventing almost all of the complications of the disease thereby reducing the reoperation rate, still retains an important role in cases with biliary or duodenal involvement [20]. Another general trend is toward minimizing, as far as possible, the metabolic sequelae of surgery in order to maintain nutritional status and provide the patient with better digestive function. In this respect, several modifications of pancreaticoduodenectomy, preserving the pylorus, have been described.

Surgical management of chronic pancreatitis remains a diffcult problem not only because of the expected end results, but also because the choice of the surgical procedure, the place of operation in the course of disease, and even the principle of operation remain actively debated.

### *Acknowledgement*

I am grateful to Mrs Jutta Gaskill for her skilful secretarial work.

### *References*

[1] Singh, S. M. and Reber, H. A. (1990). The pathology of chronic pancreatitis. *World J. Surg.*, **14**, 2–10.
[2] Sarner, M. and Cotton, P. B. (1984). Classification of pancreatitis. International Workshop, King's College, Cambridge, March 1983. *Gut*, **25**, 756–759.
[3] Maclaren, I. F. (1990). Observations and surgical management of chronic pancreatitis in the British Isles: a review of the twentieth century. *World J. Surg.*, **14**, 19–27.
[4] Nealon, W. H., Townsend, C. M. Jr. and Thompson, J. C. (1988). A comparison of large duct and small duct variants in chronic pancreatitis. *Pancreas*, **3**, 610–615.
[5] Sarles, H. and Sahel, J. (1978). Progress report: Cholestasis and lesions of the biliary tract in chronic pancreatitis. *Gut*, **19**, 851–853.
[6] Bradley, III Enteropathies, E. L. (1982). In: Complications of Pancreatitis: Medical and Surgical Management. E. L. Bradley, III, Ed. Philadelphia, *W. B. Saunders*.
[7] Salim, A. S. (1991). Role of oxygen-derived free radical scavengers in the treatment of recurrent pain produced by chronic pancreatitis. A new approach. *Arch. Surg.*, **126**, 1109–1114.
[8] Ihse, I. (1990). Pancreatic pain. *Br. J. Surg.*, **77**, 121–122.
[9] Ihse, I., Borch, K. and Larsson, J. (1990). Chronic pancreatitis: results of operations for relief of pain. *World J. Surg.*, **14**, 53–58.
[10] Okazaki, K., Yamamoto, Y. and Kagiyama, S. *et al.* (1980). Pressure of papillary zone and pancreatic main duct in patients with chronic pancreatitis in the early state. *Scand. J. Gastroenterol.*, **23**, 501–506.

[11] Huibregtse, K., Schneider, B., Vrij, A. A. and Tytgat, G. N. J. (1988). Endoscopic pancreatic drainage in chronic pancreatitis. Gastrointest. *Endosc.*, **34**, 9–15.
[12] Kozarek, R. A., Patterson, D. J., Ball, T. J. and Traverso, L. W. (1989). Endoscopic placement of pancreatic stents and drains in the management of pancreatitis. *Ann. Surg.*, **209**, 261–266.
[13] Leung, J. W. C., Browne-Wright, M., Aveling, W., Shorvon, P. J. and Cotton, P. B. (1983). Coeliac plexus block for pain in pancreatic cancer and chronic pancreatitis. *Br. J. Surg.*, **70**, 730–733.
[14] Del Maestro, R. F., Thaw, H. H., Bjork, J., Planker, M. and Arfors, K. E. (1980). Free radicals as mediators of tissue injury. *Acta. Physiol. Scand., Suppl.*, **492**, 43–57.
[15] Grisham, M. B. and Granger, D. N. (1988). Neutrophil-mediated mucosal injury: role of reactive oxygen metabolites. *Dig. Dis. Sci.*, **33**, (3rd Suppl.) 68–158.
[16] Nonaka, A., Manabe, T., Tamura, K., Asano, N., Imanishi, K. and Tobe, T. (1989). Changes in xanthine oxidase, lipid peroxide and superoxide dismutase in mouse acute pancreatitis. *Digestion*, **43**, 41–46.
[17] Sanfey, H., Bulkley, G. B. and Cameron, J. L. (1985). The pathogenesis of acute pancreatitis. The source and role of oxygen-derived free radicals in three different experimental models. *Ann. Surg.*, **201**, 633–639.
[18] Choi, T. K., Mok, F., Zhan, W. H., Fan, S. T., Lai, E. C. S. and Wong, J. (1989). Somatostatin in the treat-ment of acute pancreatitis: a prospective randomised controlled trial. *Gut*, **30**, 223–227.
[19] Beilke, M. A., Cathleen Collins-Lech and Solnle, P. G. (1987). Effect of dimethyl sulfoxide on the oxidative function of human neutrophils. *J. Lab. Clin. Med.*, **110**, 91–96.
[20] Cuilleret, J. and Guillemin, G. (1990). Surgical management of chronic pancreatitis on the continent of Europe. *World J. Surg.*, **14**, 11–18.
[21] Pain, J. A. and Knight, M. J. (1988). Pancreaticogastrostomy: The preferred operation for pain relief in chronic pancreatitis. *Br. J. Surg.*, **75**, 220–222.
[22] Gauthier-Benoit, C. and Perissat, J. (1987). Le traitement des pancréatites chroniques. Rapport au 89ème Congrès de 1' Association Francaise de Chirurgie, Paris, *Masson.*
[23] Frey, C. F., Child III, C. G. and Fry, W. (1976). Pancreatectomy for chronic pancreatitis. *Ann. Surg.*, **184**, 403–406.
[24] Beger, H. G., Krautzberger, W., Bittner, R., Büchler, M. and Limmer, J. Duodenum-preserving resection of the head of the pancreas in patients with severe chronic pancreatitis. *Surgery*, **97**, 467–470.
[25] Frey, C. F. and Smith, G. J. (1987). Description and rationale of a new operation for chronic pancreatitis. *Pancreas*, **2**, 701–706.
[26] Lambert, M. A., Linehan, J. P. and Russel, R. C. G. (1987). Duodenum preserving total pancreatectomy for end stage chronic pancreatitis. *Br. J. Surg.*, **74**, 35–38.
[27] Shires III, G. T., Warren, W. D., Millikan, W. J., Henderson, J. M. and Hersh, T. (1986). Denervated splenopancreatic flap for chronic pancreatitis. *Ann. Surg.*, **203**, 568–570.

